# Adult height is associated with increased risk of ovarian cancer: a Mendelian randomisation study

**DOI:** 10.1038/s41416-018-0011-3

**Published:** 2018-03-20

**Authors:** Suzanne C. Dixon-Suen, Christina M. Nagle, Aaron P. Thrift, Paul D. P. Pharoah, Ailith Ewing, Celeste Leigh Pearce, Wei Zheng, Australian Ovarian Cancer Study Group, Georgia Chenevix-Trench, Peter A. Fasching, Matthias W. Beckmann, Diether Lambrechts, Ignace Vergote, Sandrina Lambrechts, Els Van Nieuwenhuysen, Mary Anne Rossing, Jennifer A. Doherty, Kristine G. Wicklund, Jenny Chang-Claude, Audrey Y. Jung, Kirsten B. Moysich, Kunle Odunsi, Marc T. Goodman, Lynne R. Wilkens, Pamela J. Thompson, Yurii B. Shvetsov, Thilo Dörk, Tjoung-Won Park-Simon, Peter Hillemanns, Natalia Bogdanova, Ralf Butzow, Heli Nevanlinna, Liisa M. Pelttari, Arto Leminen, Francesmary Modugno, Roberta B. Ness, Robert P. Edwards, Joseph L. Kelley, Florian Heitz, Andreas du Bois, Philipp Harter, Ira Schwaab, Beth Y. Karlan, Jenny Lester, Sandra Orsulic, Bobbie J. Rimel, Susanne K. Kjær, Estrid Høgdall, Allan Jensen, Ellen L. Goode, Brooke L. Fridley, Julie M. Cunningham, Stacey J. Winham, Graham G. Giles, Fiona Bruinsma, Roger L. Milne, Melissa C. Southey, Michelle A. T. Hildebrandt, Xifeng Wu, Karen H. Lu, Dong Liang, Douglas A. Levine, Maria Bisogna, Joellen M. Schildkraut, Andrew Berchuck, Daniel W. Cramer, Kathryn L. Terry, Elisa V. Bandera, Sara H. Olson, Helga B. Salvesen, Liv Cecilie Vestrheim Thomsen, Reidun K. Kopperud, Line Bjorge, Lambertus A. Kiemeney, Leon F. A. G. Massuger, Tanja Pejovic, Amanda Bruegl, Linda S. Cook, Nhu D. Le, Kenneth D. Swenerton, Angela Brooks-Wilson, Linda E. Kelemen, Jan Lubiński, Tomasz Huzarski, Jacek Gronwald, Janusz Menkiszak, Nicolas Wentzensen, Louise Brinton, Hannah Yang, Jolanta Lissowska, Claus K. Høgdall, Lene Lundvall, Honglin Song, Jonathan P. Tyrer, Ian Campbell, Diana Eccles, James Paul, Rosalind Glasspool, Nadeem Siddiqui, Alice S. Whittemore, Weiva Sieh, Valerie McGuire, Joseph H. Rothstein, Steven A. Narod, Catherine Phelan, Harvey A. Risch, John R. McLaughlin, Hoda Anton-Culver, Argyrios Ziogas, Usha Menon, Simon A. Gayther, Susan J. Ramus, Aleksandra Gentry-Maharaj, Anna H. Wu, Malcolm C. Pike, Chiu-Chen Tseng, Jolanta Kupryjanczyk, Agnieszka Dansonka-Mieszkowska, Agnieszka Budzilowska, Iwona K. Rzepecka, Penelope M. Webb, on behalf of the Ovarian Cancer Association Consortium

**Affiliations:** 1 Gynaecological Cancers Group, QIMR Berghofer Medical Research Institute, 300 Herston Road, Brisbane, QLD 4006 Australia; 2 The University of Queensland, School of Public Health, Level 2 Public Health Building (887), Corner of Herston Road & Wyndham Street, Brisbane, QLD 4006 Australia; 3 Department of Medicine and Dan L Duncan Comprehensive Cancer Center, Baylor College of Medicine, One Baylor Plaza, Houston, TX 77030 USA; 4 Strangeways Research Laboratory, Centre for Cancer Genetic Epidemiology, Department of Public Health & Primary Care/Department of Oncology, University of Cambridge, Worts Causeway, Cambridge, CB1 8RN UK; 5 Department of Epidemiology, University of Michigan School of Public Health, 1415 Washington Heights, SPH Tower, Ann Arbor, MI 48109-2029 USA; 6 Department of Preventive Medicine, Keck School of Medicine, University of Southern California Norris Comprehensive Cancer Center, 1441 Eastlake Avenue, Los Angeles, CA 90033 USA; 7 Vanderbilt Epidemiology Center, Vanderbilt University School of Medicine, 2525 West End Ave., Nashville, TN 37203 USA; 8 Cancer Genetics and Genomics Laboratory, Peter MacCallum Cancer Centre, St Andrews Place, Melbourne, VIC 3002 Australia; 9 Cancer Genetics Group, QIMR Berghofer Medical Research Institute, 300 Herston Road, Brisbane, QLD 4006 Australia; 10 Department of Medicine, Division of Hematology and Oncology, David Geffen School of Medicine, University of California at Los Angeles, 10833 Le Conte Ave, Los Angeles, CA 90095 USA; 11 Department of Gynecology and Obstetrics, University Hospital Erlangen, Friedrich-Alexander-University Erlangen-Nuremberg, Comprehensive Cancer Center Erlangen-EMN, Universitätsstrasse 21-23, 91054 Erlangen, Germany; 12 Vesalius Research Center, VIB, Herestraat 49, bus 912, 3000 Leuven, Belgium; 13 Laboratory for Translational Genetics, Department of Oncology, University of Leuven, O&N IV Herestraat 49—Box 912, 3000 Leuven, Belgium; 14 Division of Gynecologic Oncology, Department of Obstetrics and Gynaecology and Leuven Cancer Institute, University Hospitals Leuven, Herestraat 49, Leuven, 3000 Belgium; 15 Program in Epidemiology, Division of Public Health Sciences, Fred Hutchinson Cancer Research Center, 1100 Fairview Ave. N., Seattle, WA 98109-1024 USA; 16 Department of Epidemiology, University of Washington, 1959 NE Pacific Street, Health Sciences Bldg, F-262, Seattle, WA 98195 USA; 17 Department of Epidemiology, The Geisel School of Medicine at Dartmouth, 1 Medical Center Drive, 7927 Rubin Building, Lebanon, NH 03756 USA; 18 Division of Cancer Epidemiology, German Cancer Research Center, Im Neuenheimer Feld 581, Heidelberg, 69120 Germany; 19 University Cancer Center Hamburg, University Medical Center Hamburg-Eppendorf, Martinistr. 52, 20246 Hamburg, Germany; 20 Department of Cancer Prevention and Control, Roswell Park Cancer Institute, Elm and Carlton Streets, Buffalo, NY 14263 USA; 21 Department of Gynecological Oncology, Roswell Park Cancer Institute, Elm and Carlton Streets, Buffalo, NY 14263 USA; 22 Cancer Prevention and Control, Samuel Oschin Comprehensive Cancer Institute, Cedars-Sinai Medical Center, 8700 Beverly Blvd., Los Angeles, CA 90048 USA; 23 Community and Population Health Research Institute, Department of Biomedical Sciences, Cedars-Sinai Medical Center, 8700 Beverly Blvd., Los Angeles, CA 90048 USA; 24 Cancer Epidemiology Program, University of Hawaii Cancer Center, 701 Ilalo Street, Honolulu, HI 96813 USA; 25 Clinics of Obstetrics and Gynaecology, Hannover Medical School, Carl-Neuberg-Str. 1, D-30625 Hannover, Germany; 26 Radiation Oncology Research Unit, Hannover Medical School, Carl-Neuberg-Str. 1, D-30625 Hannover, Germany; 27 Department of Pathology, University of Helsinki and Helsinki University Hospital, Haartmaninkatu 8, 00029 Helsinki, Finland; 28 Department of Obstetrics and Gynecology, University of Helsinki and Helsinki University Hospital, Haartmaninkatu 8, 00029 Helsinki, Finland; 29 Division of Gynecologic Oncology, Department of Obstetrics, Gynecology and Reproductive Sciences, University of Pittsburgh School of Medicine, 300 Halket Street, Pittsburgh, PA 15213 USA; 30 Ovarian Cancer Center of Excellence, Women’s Cancer Research Program, Magee-Women’s Research Institute and University of Pittsburgh Cancer Institute, 204 Craft Avenue, Pittsburgh, PA 15213 USA; 31 Department of Epidemiology, University of Pittsburgh Graduate School of Public Health, 130 De Soto Street, Pittsburgh, PA 15261 USA; 32 The University of Texas Health Science Center at Houston, School of Public Health, 1200 Herman Pressler, Suite E-1015, Houston, TX 77030 USA; 33 Department of Gynecology and Gynecologic Oncology, Kliniken Essen-Mitte/ Evang. Huyssens-Stiftung/ Knappschaft GmbH, Henricistrasse 92, 45136 Essen, Germany; 34 Department of Gynecology and Gynecologic Oncology, Dr. Horst Schmidt Kliniken Wiesbaden, Ludwig-Erhard-Strasse 100, 65199 Wiesbaden, Germany; 35 Praxis für Humangenetik, Biebricher Allee 117, 65187 Wiesbaden, Germany; 36 Women’s Cancer Program at the Samuel Oschin Comprehensive Cancer Institute, Cedars-Sinai Medical Center, 8635 West Third Street, Los Angeles, CA 90048 USA; 37 Department of Virus, Lifestyle and Genes, Danish Cancer Society Research Center, Strandboulevarden 49, DK-2100 Copenhagen, Denmark; 38 Department of Gynaecology, Rigshospitalet, University of Copenhagen, Blegdamsvej 9, DK-2100 Copenhagen, Denmark; 39 Molecular Unit, Department of Pathology, Herlev Hospital, University of Copenhagen, Herlev Ringvej 75, DK-2370 Herlev, Denmark; 40 Department of Health Science Research, Division of Epidemiology, Mayo Clinic, 200 First Street SW, Charlton 6, Rochester, MN 55905 USA; 41 Department of Biostatistics and Bioinformatics, Moffitt Cancer Center, 12902 Magnolia Drive, Tampa, FL 33612 USA; 42 Department of Laboratory Medicine and Pathology, Mayo Clinic, 200 First Street SW, Stabile 13, Rochester, MN 55905 USA; 43 Division of Biomedical Statistics and Informatics, Department of Health Science Research, Mayo Clinic, 200 First Street SW, Charlton 6, Rochester, MN 55905 USA; 44 Cancer Epidemiology and Intelligence Division, Cancer Council Victoria, 615 St Kilda Road, Melbourne, VIC 3004 Australia; 45 Centre for Epidemiology and Biostatistics, Melbourne School of Population and Global Health, The University of Melbourne, Grattan Street, Parkville, VIC 3010 Australia; 46 Department of Epidemiology and Preventive Medicine, Monash University, The Alfred Centre, 99 Commercial Road, Melbourne, VIC 3004 Australia; 47 Genetic Epidemiology Laboratory, Department of Pathology, The University of Melbourne, Grattan Street, Carlton, VIC 3053 Australia; 48 Department of Epidemiology, The University of Texas MD Anderson Cancer Center, 1155 Pressler Blvd—Unit 1340, Houston, TX 77030 USA; 49 Department of Gynecologic Oncology, The University of Texas MD Anderson Cancer Center, 1155 Pressler Blvd - Unit 1340, Houston, TX 77030 USA; 50 College of Pharmacy and Health Sciences, Texas Southern University, 3100 Cleburne St, Houston, TX 77004 USA; 51 Division of Gynecologic Oncology, Department of Obstetrics And Gynecology, NYU Langone Medical Center, 240 East 38th Street, New York, NY 10016 USA; 52 Gynecology Service, Department of Surgery, Memorial Sloan Kettering Cancer Center, 417 East 68th Street, New York, NY 10065 USA; 53 Department of Public Health Sciences, The University of Virginia, Box 800717, Charlotteville, VA 22908 USA; 54 Department of Obstetrics and Gynecology, Duke University Medical Center, 25171 Morris Bldg, Durham, NC 27710 USA; 55 Obstetrics and Gynecology Epidemiology Center, Brigham and Women’s Hospital, 221 Longwood Avenue, Richardson Fuller Building, Boston, MA 02115 USA; 56 Department of Epidemiology, Harvard T.H. Chan School of Public Health, 677 Huntington Ave, Boston, MA 02115 USA; 57 Cancer Prevention and Control Program, Rutgers Cancer Institute of New Jersey, 195 Little Albany Street, New Brunswick, NJ 08903 USA; 58 Rutgers School of Public Health, 683 Hoes Lane West, Piscataway, NJ 08854 USA; 59 Department of Epidemiology and Biostatistics, Memorial Sloan Kettering Cancer Center, 485 Lexington Ave, New York, NY 10017 USA; 60 Department of Obstetrics and Gynecology, Haukeland University Hospital, Kvinneklinikken, Jonas Liesvei 72, 5058 Bergen, Norway; 61 Centre for Cancer Biomarkers, Department of Clinical Science, University of Bergen, Postboks 7804, N-5020 Bergen, Norway; 62 Radboud University Medical Center, Radboud Institute for Health Sciences, PO Box 9101, 6500 HB Nijmegen, The Netherlands; 63 Radboud University Medical Center, Radboud Institute for Molecular Life Sciences, Department of Gynaecology, PO Box 9101, 6500 HB Nijmegen, The Netherlands; 64 Department of Obstetrics & Gynecology, Oregon Health & Science University, 3181 SW Sam Jackson Park Road, Portland, OR 97239 USA; 65 Knight Cancer Institute, Oregon Health & Science University, 3181 SW Sam Jackson Park Road, Portland, OR 97239 USA; 66 Division of Epidemiology and Biostatistics, Department of Internal Medicine, University of New Mexico, 2703 Frontier Ave NE, Albuquerque, NM 87131 USA; 67 Cancer Control Research, BC Cancer Agency, 675 West 10th Avenue, Vancouver, BC Canada; 68 Department of Medicine, University of British Columbia, 2775 Laurel Street, Vancouver, BC V5Z 1M9 Canada; 69 Canada’s Michael Smith Genome Sciences Centre, BC Cancer Agency, 675 West 10th Avenue, Vancouver, BC Canada; 70 Department of Biomedical Physiology and Kinesiology, Simon Fraser University, 8888 University Drive, Burnaby, BC V5A 1S6 Canada; 71 Department of Public Health Sciences, Medical University of South Carolina, 68 President Street, Bioengineering Building, Charleston, SC 29425 USA; 72 International Hereditary Cancer Center, Department of Genetics and Pathology, Pomeranian Medical University, ul. Połabska 4, Szczecin, 70-115 Poland; 73 Department of Gynecological Surgery and Gynecological Oncology of Adults and Adolescents, Pomeranian Medical University, ul. Powstańców Wlkp 72, 70-111 Szczecin, Poland; 74 Division of Cancer Epidemiology and Genetics, National Cancer Institute, 9609 Medical Center Drive, Rockville, MD 20850 USA; 75 Department of Cancer Epidemiology and Prevention, The Maria Sklodowska-Curie Memorial Cancer Center and Institute of Oncology, Wawelska 15B, 02-034 Warsaw, Poland; 76 Cancer Genetics Laboratory, Research Division, Peter MacCallum Cancer Centre, St Andrews Place, Melbourne, VIC 3002 Australia; 77 Department of Pathology, University of Melbourne, Grattan Street, Carlton, VIC 3053 Australia; 78 Faculty of Medicine, Southampton University Hospitals Trust, Princess Anne Hospital, University of Southampton, Southampton, SO16 5YA UK; 79 Cancer Research UK Clinical Trials Unit Glasgow, Institute of Cancer Sciences, University of Glasgow, 1053 Gt. Western Road, Glasgow, G12 0YN UK; 80 The Beatson West of Scotland Cancer Centre, 1053 Gt. Western Road, Glasgow, G12 0YN UK; 81 Department of Gynaecological Oncology, Glasgow Royal Infirmary, Alexandra Parade, Glasgow, G31 2ER UK; 82 Department of Health Research and Policy—Epidemiology, Stanford University School of Medicine, HRP Redwood Building, 259 Campus Drive, Stanford, CA 94305 USA; 83 Departments of Population Health Science & Policy and Genetics & Genomic Sciences, Icahn School of Medicine at Mount Sinai, 1 Gustave L. Levy Place, New York, NY 10029 USA; 84 Women’s College Research Institute, University of Toronto, 790 Bay Street, Toronto, ON M5G 1N8 Canada; 85 Department of Cancer Epidemiology, Moffitt Cancer Center, 12902 Magnolia Drive, Tampa, FL 33612 USA; 86 Department of Chronic Disease Epidemiology, Yale School of Public Health, LEPH 413, 60 College Street, New Haven, CT 06510 USA; 87 Public Health Ontario, 480 University Avenue (/300), Toronto, ON M5G1V2 Canada; 88 Department of Epidemiology, University of California Irvine, 224 Irvine Hall, Irvine, CA 92697-7550 USA; 89 Genetic Epidemiology Research Institute, UCI Center for Cancer Genetics Research & Prevention, School of Medicine, University of California Irvine, 224 Irvine Hall, Irvine, CA 92697-7550 USA; 90 Women’s Cancer, Institute for Women’s Health, University College London, Maple House 1st Floor, 149 Tottenham Court Road, London, W1T 7DN UK; 91 Center for Cancer Prevention and Translational Genomics, Samuel Oschin Cancer Institute, Spielberg Building, 8725 Alden Dr., Los Angeles, CA 90048 USA; 92 School of Women’s and Children’s Health, University of New South Wales, Level 1, Women’s Health Institute, Royal Hospital for Women, Barker Street, Randwick, NSW 2031 Australia; 93 The Kinghorn Cancer Centre, Garvan Institute of Medical Research, 384 Victoria Street, Darlinghurst, NSW 2010 Australia; 94 Department of Pathology and Laboratory Diagnostics, The Maria Sklodowska-Curie Memorial Cancer Center and Institute of Oncology, Roentgena 5, 02-781 Warsaw, Poland

**Keywords:** Genetics research, Ovarian cancer, Cancer epidemiology, Risk factors

## Abstract

**Background:**

Observational studies suggest greater height is associated with increased ovarian cancer risk, but cannot exclude bias and/or confounding as explanations for this. Mendelian randomisation (MR) can provide evidence which may be less prone to bias.

**Methods:**

We pooled data from 39 Ovarian Cancer Association Consortium studies (16,395 cases; 23,003 controls). We applied two-stage predictor-substitution MR, using a weighted genetic risk score combining 609 single-nucleotide polymorphisms. Study-specific odds ratios (OR) and 95% confidence intervals (CI) for the association between genetically predicted height and risk were pooled using random-effects meta-analysis.

**Results:**

Greater genetically predicted height was associated with increased ovarian cancer risk overall (pooled-OR (pOR) = 1.06; 95% CI: 1.01–1.11 per 5 cm increase in height), and separately for invasive (pOR = 1.06; 95% CI: 1.01–1.11) and borderline (pOR = 1.15; 95% CI: 1.02–1.29) tumours.

**Conclusions:**

Women with a genetic propensity to being taller have increased risk of ovarian cancer. This suggests genes influencing height are involved in pathways promoting ovarian carcinogenesis.

## Introduction

Observational studies have reported a positive association between adult height and ovarian cancer risk.^1–4^ However, these studies were subject to the biases inherent in conventional observational studies, including selection bias, differential and non-differential reporting bias and confounding. The degree to which these factors could account for the observed association is uncertain. Mendelian randomisation (MR) uses genetic markers as proxies for environmental exposures and, due to the singular qualities of genotype data, can provide complementary evidence by overcoming many biases affecting conventional studies.^5^ We used MR to examine the relationship between height and ovarian cancer risk in the Ovarian Cancer Association Consortium (OCAC),^6^ aiming to provide more certainty about the relationship between height and ovarian cancer risk. We hypothesised that greater genetically predicted height would be associated with increased risk.

## Materials and methods

### Study population and outcomes

We pooled data from 16,395 genetically European women with primary ovarian/fallopian tube/peritoneal cancer and 23,003 controls from 39 OCAC studies (Table 1; Supplementary Table 1). The data set and methods have been described previously.^7^ Participants were genotyped via the Collaborative Oncological Gene-Environment Study.^8^ Twenty-two studies provided height data (16 provided parity, oral contraceptive (OC) use, education and age at menarche information) for >50% of their participants. We first considered all cases, then stratified by tumour behaviour. Secondary analyses stratified by histologic subtype/behaviour.

**Table 1:** Characteristics of 39 OCAC studies and 39,398 participants of European ancestry included in the Mendelian randomisation analysis

Study acronym^a^	Country	Diagnosis (years)	Median (range) age at diagnosis/interview	Invasive cases (*N*)	Borderline cases (*N*)	All cases (*N*)^b^	Controls (*N*)	Mean (SD) height (cm)^c^
AUS	Australia	2002–2006	58 (19–80)	859	1	860	977	163 (6.9)
BAV	Germany	2002–2008	58 (24–83)	96	5	102	143	164 (5.8)
BEL	Belgium	2007–2010	46 (19–87)	275	0	275	1347	—
DOV	USA	2002–2009	57 (35–74)	904	327	1231	1487	166 (6.5)
GER	Germany	1993–1998	57 (21–75)	189	24	213	413	163 (6.0)
GRR	USA	1981–2012	48 (21–83)	125	0	125	0	—
HAW	USA	1993–2008	56 (27–87)	60	20	80	157	163 (6.6)
HJO	Germany	2007–2011	54 (18–88)	261	13	290	273	—
HMO	Belarus	2006–2011	45 (22–76)	142	0	143	138	—
HOC	Finland	1975–1999	46 (18–86)	210	8	239	447	—
HOP	USA	2003–2009	58 (25–94)	567	71	723	1464	163 (6.8)
HSK	Germany	2000–2007	58 (18–81)	147	9	156	0	165 (5.6)
LAX	USA	1989–2008	58 (31–88)	278	0	278	0	—
MAL	Denmark	1994–1999	57 (31–80)	440	138	578	828	166 (6.1)
MAY	USA	2000–2010	61 (20–93)	699	79	778	743	165 (6.3)
MCC	Australia	1990–2008	65 (45–79)	66	0	66	66	159 (7.0)
MDA	USA	1997–2009	62 (23–88)	375	0	375	384	—
MSK	USA	1997–2010	57 (18–89)	450	0	450	593	—
NCO	USA	1999–2008	57 (20–75)	722	171	896	792	163 (6.4)
NEC	USA	1992–2003	52 (21–78)	654	232	904	1009	163 (6.7)
NJO	USA	2002–2009	60 (25–88)	169	0	169	181	163 (6.9)
NOR	Norway	2001–2010	51 (18–86)	236	12	248	371	—
NTH	Netherlands	1997–2008	55 (18–83)	292	3	295	323	167 (6.0)
ORE	USA	2007–2011	58 (22–86)	55	9	65	0	—
OVA	Canada	2002–2009	58 (19–80)	640	161	801	748	—
POC	Poland	1998–2008	55 (23–82)	423	0	423	417	—
POL	Poland	2000–2004	56 (24–74)	236	0	236	223	162 (5.6)
PVD	Denmark	2004–2009	63 (30–88)	168	0	168	0	165 (6.5)
RMH	UK	1993–1996	52 (26–73)	148	7	155	0	—
SEA	UK	1998–2011	57 (19–78)	1447	76	1530	6004	162 (6.3)
SOC	UK	1993–1998	62 (22–92)	268	20	288	0	—
SRO	UK	1999–2001	59 (34–84)	158	0	158	0	—
STA	USA	1997–2002	50 (20–64)	251	10	261	313	165 (6.7)
TOR	Canada	1995–2007	58 (26–85)	603	0	605	440	163 (7.1)
UCI	USA	1993–2005	56 (18–86)	277	141	418	367	165 (6.6)
UKO	UK	2006–2010	63 (19–89)	718	0	718	1104	162 (6.7)
UKR	UK	1991–2009	54 (24–77)	47	0	47	0	—
USC	USA	1992–2010	57 (22–82)	693	152	845	1047	165 (6.8)
WOC	Poland	1997–2010	44 (20–81)	201	2	203	204	—

All participants were of >90% European ancestry according to genetic markers of ancestry.

^a^OCAC is an international collaboration of largely case–control studies. See Supplementary Table 1 for study names and references. To maximise power, nine case-only studies were grouped for analysis with case–control studies from the same region: HSK combined with GER; GRR with HOP; PVD with MAL; RMH, SOC, SRO, UKR with SEA and UKO (‘UK group’); ORE with DOV; LAX with UCI.

^b^Cases had primary ovarian (*n* = 15,636), fallopian tube (*n* = 180) or peritoneal (*n* = 552) cancer or ovarian/tubal/peritoneal tumours of undetermined site (*n* = 27).

^c^Usual adult height. Height is summarised for 22 studies (20 case–control studies) where >50% participants had data available (AUS, BAV, DOV, GER, HAW, HOP, HSK, MAL, MAY, MCC, NCO, NEC, NJO, NTH, POL, PVD, SEA, STA, TOR, UCI, UKO, USC). Sixteen of these 22 studies were also used in conventional height analyses, as they provided data on potential confounders (age, parity, use of oral contraceptives, education, and age at menarche) for >50% of participants (AUS, DOV, GER, HAW, HOP, MAL, NCO, NEC, NJO, NTH, POL, STA, TOR, UCI, UKO, USC).

*OCAC* Ovarian Cancer Association Consortium, *SD* standard deviation

### Genetic risk score

The Genetic Investigation of ANthropometric Traits (GIANT) Consortium had previously identified 697 single-nucleotide polymorphisms (SNPs) significantly associated with height.^9^ In our sample, 92 of these SNPs had been genotyped and the remainder were imputed using 1000 Genome Project data.^8, 10^ After excluding poorly-imputed SNPs (quality *r*^2^ < 0.6), 609 remained (92 genotyped/517 imputed) (Supplementary Table 2). In controls, minor allele frequencies (MAFs) were >5% (except for 16 SNPs, MAFs 1.7–4.9%).

We constructed a weighted genetic risk score (GRS) for height by summing height-increasing alleles across the 609 SNPs (‘GRS-609’/‘the GRS’), weighting alleles by *β*-coefficients for their association with height reported by GIANT. The score represents predicted additional height conferred by these variants, compared to having no height-increasing alleles. We report results for 5 cm increments.

### Statistical analysis

Statistical methods have been described previously.^7^ Briefly, we used individual-level OCAC data for two-stage predictor-substitution MR ^11, 12^: first, we predicted height from the weighted GRS for all participants using coefficients from linear regression in 17,649 controls with height data; second, within each study, we used logistic regression to model disease status on GRS-predicted height. Models adjusted for age and five principal components for population structure.^8^ We combined study-specific estimates using meta-analysis,^13^ generating pooled odds ratios (pOR) and 95% confidence intervals (CI) for the trend in risk per 5 cm increase in predicted height. We had 97% power to detect an OR of 1.10 (mRnd tool).^14^

Sensitivity analyses included removing 16 SNPs with MAFs <5%, and restricting to SNPs with imputation *r*^2^ ≥ 0.9 (‘GRS-363’), SNPs representing distinct loci^9^ (‘GRS-377’), and directly-genotyped SNPs (‘GRS-92’). We examined whether potential confounders of the association in observational studies were associated with the GRS. To assess robustness to pleiotropy (where SNPs may influence risk via pathways not mediated through height), we conducted MR-Egger regression^15^ and assessed smaller GRSs excluding SNPs with the highest probability of acting via other pathways from GRS to outcome (SNPs associated with ovarian/other hormonal cancers (breast, prostate), hormone levels and in/near tumour initiation/growth genes). We identified these potentially pleiotropic, pathway-specific SNPs via the NHGRI GWAS Catalog,^16^ the UCSC Genome/Table Browsers^17, 18^ and from lists of SNPs nominated for iCOGS genotyping by ovarian, breast and prostate cancer researchers (to capture SNPs of interest unpublished at the time of analysis).

Secondary analyses defined cases by histologic subtype/behaviour. Among 16 studies with height/confounder data, we conducted conventional analysis (adjusted for parity, OC use, education, menarche age; stratified by study, 5-year age group) and compared results with MR-estimates among the same women.

Analyses were performed using SAS 9.2 (SAS Institute Inc., Cary, NC) and Stata 13.0 (StataCorp LP, College Station, TX). This work and each contributing study was approved by the appropriate institutional review board/ethics committee. All participants provided informed consent.

## Results

### Population characteristics

We included 16,395 cases (14,549 invasive tumours, 1691 borderline, 155 of unknown behaviour) and 23,003 controls (Table 1). The median diagnosis year was 2003, with 74% diagnosed post-2000. Participants were aged 18–94 (median 56) years at diagnosis/interview. Mean height ranged from 159 to 167 cm across 22 studies with data, and was 163 (standard error (SE) = 0.05) cm for controls and 164 (SE = 0.06) cm for cases (*p* < 0.0001).

### Genetic risk score characteristics

The GRS-609 was normally distributed in controls, ranging from 15.45 to 18.99 (median = 17.23; interquartile range = 16.92–17.54). It explained 13% of variance in height, 17% after adjusting for age and principal components (partial-*R*^2^ = 12%; first-stage regression partial-*F*-statistic = 2505.8 (df = 1), *p* < 0.001). A 1-unit GRS-609 increase was associated with 5.2 cm greater height. Average height was 6.2 cm greater in the highest vs. lowest GRS quartile.

Cochran’s *I*^2^ and *p*-values for heterogeneity^19^ showed no evidence of inter-study heterogeneity in the relationship between either the GRS-609 (*I*^2^ = 34%, *p*-heterogeneity = 0.07) or the simplified GRS-363 (*I*^2^ = 32%, *p*-heterogeneity = 0.08) and height among controls (Supplementary Figure 1a, b). The GRS-609 was not associated with most potential confounders of the height-ovarian cancer association in observational studies, including age, parity, OC use and education (Supplementary Table 3). The GRS was marginally positively associated with age at menarche (*p* = 0.03), consistent with known genetic overlap between these traits.^20^

### Primary outcomes

**Fig. 1:**
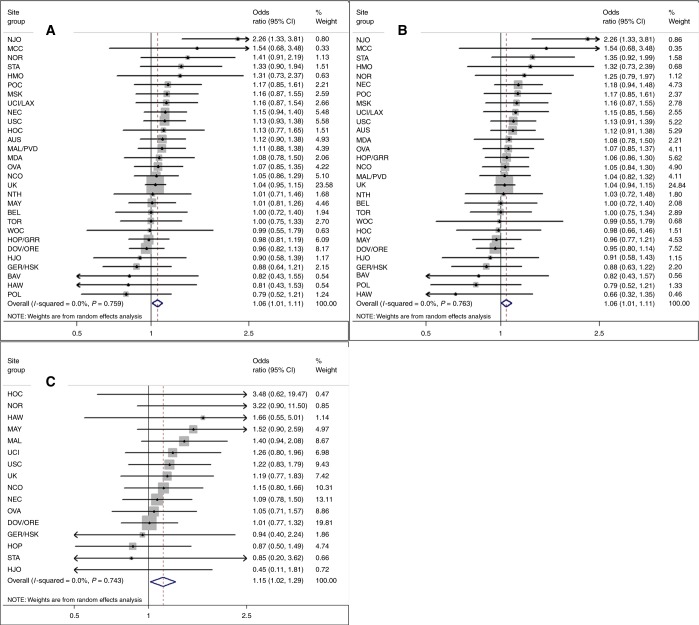
Association between increasing genetically predicted height and risks of all, invasive and borderline ovarian tumours. Increasing height per 5 cm predicted by weighted 609-locus genetic risk score among 39 studies. Risk of **a** all, **b** invasive and **c** borderline ovarian tumours. The UK grouping includes RMH, SOC, SRO, UKR, SEA and UKO for **a** and **b**, and RMH, SOC and SEA for **c**

Women with greater genetically predicted height had a modestly increased risk of developing ovarian cancer (pOR = 1.06, 95% CI: 1.01–1.11 per 5 cm) (Fig. 1a; Table 2) with a greater magnitude of association for borderline (pOR = 1.15; 95% CI: 1.02–1.29) than invasive tumours (pOR = 1.06; 95% CI: 1.01–1.11; Fig. 1b, c; Table 2). No significant inter-study heterogeneity was noted (Fig. 1a–c). GRS-363 (pOR = 1.06, 95% CI: 1.00–1.11, all tumours) and GRS-377 (OR = 1.07; 95% CI: 1.01–1.12) results were similar to the GRS-609. The association was stronger when we restricted to 92 genotyped SNPs (pOR = 1.14; 95% CI: 1.04–1.25). Estimates from analyses excluding low-MAF SNPs, excluding case-only studies, or adjusting for age at menarche, were similar to primary analyses. When we sequentially excluded SNPs associated with ovarian or other hormonal cancers, hormone levels and tumour development, estimates were similar or stronger than GRS-609 results. MR-Egger suggested minimal bias from pleiotropy (*p* = 0.1; MR-Egger beta = 0.163 corresponded to an OR per 5 cm of 1.13 (95% CI: 1.02–1.25), confirming a significant positive association).

**Table 2:** Association between increasing height (per 5 cm)—predicted by a weighted 609-locus genetic risk score—and risk of ovarian cancer, stratified by study

Histologic subtype^a^	*N* studies	*N* controls	*N* cases	Odds ratios (95% CI)^b^
Primary outcomes				
All ovarian cancers	39	23,003	16,395	1.06 (1.01–1.11)
Invasive	39	23,003	14,549	1.06 (1.01–1.11)
Borderline^c^	20	16,463	1680	1.15 (1.02–1.29)
Secondary outcomes, by histologic subtype and behaviour				
Serous				
High-grade^d^	39	23,003	7933	1.05 (0.99–1.11)
Invasive low-grade and borderline	32	21,131	1408	1.15 (1.01–1.30)
Mucinous (invasive and borderline)	38	22,410	1567	1.08 (0.96–1.21)
Endometrioid (invasive)	39	23,003	2059	1.05 (0.95–1.16)
Clear cell (invasive)	35	22,051	948	1.20 (1.04–1.38)

Weights applied were *β*-coefficients for the relationship between each SNP and height as reported in the meta-analysis of genome-wide association studies conducted by the Genetic Investigation of ANthropometric Traits (GIANT) Consortium.^9^ On the basis of the additive SNP effects suggested by GIANT, the score summed alleles across the 609 SNPs. For the 92 genotyped SNPs, where values were missing (<2.5% per SNP), we used imputed probabilities.

^a^Includes studies with >5 cases.

^b^Pooled study-specific odds ratios are reported for primary outcomes; odds ratios stratified by study are reported for secondary outcomes (secondary analyses used single models stratified by study to maximise power).

^c^Of the 1691 borderline tumours included in the all-case analysis, 1680 were from 20 studies with >5 cases each.

^d^Includes all invasive serous cancers except low-grade (G1) (*n* = 469) as well as invasive serous cancers of unknown grade (*n* = 1957) and primary peritoneal cancers of unknown behaviour (*n* = 44), because in both instances the majority would be high-grade serous.

*CI* confidence interval

In contrast, for women with height and confounder data (16 studies), the conventional analysis suggested no association (adjusted-OR = 1.01, 95% CI: 0.99–1.04 per 5 cm). Conducting MR within the same 16 studies yielded results similar to overall analyses (OR = 1.06, 95% CI: 1.00–1.13) (Supplementary Table 4).

### Secondary outcomes

After stratifying by subtype/behaviour, the strongest associations were seen for clear cell (OR = 1.20, 95% CI: 1.04–1.38) and low-grade/borderline serous cancers (OR = 1.15, 95% CI: 1.01–1.30) (Table 2). However, CIs were wide and overlapping due to lower statistical power in these subgroup analyses. The estimate for clear cell cancers was also significantly elevated in our conventional analyses (Supplementary Table 4).

## Discussion

We used a 609-SNP GRS to examine the relationship between height and ovarian cancer risk for women of European ancestry. Our data indicate a modest positive association between genetically predicted height and ovarian cancer risk, which may be stronger for borderline cancers. Height may be relevant to cancer risk as a marker for lifetime growth-factor levels (e.g. IGF-1) and/or early-life exposures (socio-economic/environmental/nutritional).^3, 21, 22^

Observational studies are subject to biases (reverse causality, selection bias, differential/non-differential reporting, confounding) which cannot be ruled out as possible explanations for observed associations. By using genotype, the MR technique can overcome some of these biases, given three assumptions. We confirmed the two verifiable assumptions: the GRS was associated with height, and not with most known confounders. The GRS-menarche age association is unlikely to explain the observed association, because age at menarche is only weakly associated with ovarian cancer, and women with later menarche have if anything lower ovarian cancer risk, so if this affected our results, we would expect the true effect to be at least as strong as the reported association. Also, removing hormone-related SNPs, or adjusting for menarche age, did not attenuate estimates. Owing to the limited current biological understanding of all 609 SNPs, we could not conclusively exclude the presence of alternate pathways from height genes to ovarian cancer (assumption three). However, MR-Egger and sensitivity analyses excluding pathway-specific SNPs provided some evidence for their absence, minimising the likelihood that our observed association is explained by pathways separate from height/growth. Although height data were not available for the entire population, this is unlikely to have affected our results as we used these data only to refine the height predictions from the GRS, and there is no reason to believe the GRS-height relationship would be different for women with and without height data. Further strengths of our analysis include the large number of SNPs and power to detect modest differences.

Despite potential limitations of conventional observational studies, our MR-estimate is almost identical to previously reported associations, suggesting previous estimates were not appreciably biased. The World Cancer Research Fund/American Institute for Cancer Research meta-analysis of 24 prospective studies, and a study pooling 47 prospective/case–control studies, both reported a significant 7–8% increase in risk (combining invasive/borderline cancers) per 5 cm height increase.^3, 4^ The lack of association seen in the OCAC conventional height analysis reflects the greater potential for bias in case–control studies and illustrates the value of MR in overcoming these biases. Few previous studies have examined borderline cancers separately, a strength of our analysis. Previous observational studies have not reported consistent patterns by histologic subtype^2, 4, 23^; our secondary analyses were under-powered to resolve this question.

Using MR, we have established that the previously observed association between height and ovarian cancer risk is unlikely to have been explained by bias, and that genetic factors influencing height play roles in ovarian cancer development. Height could therefore be used, with other risk factors, to identify women at elevated risk. Further research should continue to explore mechanisms underpinning this association.

## Electronic supplementary material

Supplementary Information
