# Supplementary material for: Adult height is associated with increased risk of ovarian cancer: a Mendelian randomisation study
Source: Br J Cancer. 2018 Mar 20;118(8):1123–9. doi: 10.1038/s41416-018-0011-3 (PMC5931085; doi:10.1038/s41416-018-0011-3)
Supplement: Supplementary file 1 — Supplementary Information [file 41416_2018_11_MOESM1_ESM.docx]

# Supplementary Information

**Adult height is associated with increased risk of ovarian cancer: a Mendelian Randomization study**

**British Journal of Cancer**

Suzanne C. Dixon-Suen, Christina M. Nagle, Aaron P. Thrift, Paul D.P. Pharoah, Ailith Pirie, Celeste Leigh Pearce, Wei Zheng, Australian Ovarian Cancer Study Group, Georgia Chenevix-Trench, Peter A. Fasching, Matthias W. Beckmann, Diether Lambrechts, Ignace Vergote, Sandrina Lambrechts, Els Van Nieuwenhuysen, Mary Anne Rossing, Jennifer A. Doherty, Kristine G. Wicklund, Jenny Chang-Claude, Audrey Y. Jung, Kirsten B. Moysich, Kunle Odunsi, Marc T. Goodman, Lynne R. Wilkens, Pamela J. Thompson, Yurii B. Shvetsov, Thilo Dörk, Tjoung-Won Park-Simon, Peter Hillemanns, Natalia Bogdanova, Ralf Butzow, Heli Nevanlinna, Liisa M. Pelttari, Arto Leminen, Francesmary Modugno, Roberta B. Ness, Robert P. Edwards, Joseph L. Kelley, Florian Heitz, Andreas du Bois, Philipp Harter, Ira Schwaab, Beth Y. Karlan, Jenny Lester, Sandra Orsulic, Bobbie J. Rimel, Susanne K. Kjær, Estrid Høgdall, Allan Jensen, Ellen L. Goode, Brooke L. Fridley, Julie M. Cunningham, Stacey J. Winham, Graham G. Giles, Fiona Bruinsma, Roger L. Milne, Melissa C. Southey, Michelle A.T. Hildebrandt, Xifeng Wu, Karen H. Lu, Dong Liang, Douglas A. Levine, Maria Bisogna, Joellen M. Schildkraut, Andrew Berchuck, Daniel W. Cramer, Kathryn L. Terry, Elisa V. Bandera, Sara H. Olson, Helga B. Salvesen, Liv Cecilie Vestrheim Thomsen, Reidun K. Kopperud, Line Bjorge, Lambertus A. Kiemeney, Leon F.A.G. Massuger, Tanja Pejovic, Amanda Bruegl, Linda S. Cook, Nhu D. Le, Kenneth D. Swenerton, Angela Brooks-Wilson, Linda E. Kelemen, Jan Lubiński, Tomasz Huzarski, Jacek Gronwald, Janusz Menkiszak, Nicolas Wentzensen, Louise Brinton, Hannah Yang, Jolanta Lissowska, Claus K. Høgdall, Lene Lundvall, Honglin Song, Jonathan P. Tyrer, Ian Campbell, Diana Eccles, James Paul, Rosalind Glasspool, Nadeem Siddiqui, Alice S. Whittemore, Weiva Sieh, Valerie McGuire, Joseph H. Rothstein, Steven A. Narod, Catherine Phelan, Harvey A. Risch, John R. McLaughlin, Hoda Anton-Culver, Argyrios Ziogas, Usha Menon, Simon A. Gayther, Susan J. Ramus, Aleksandra Gentry-Maharaj, Anna H. Wu, Malcolm C. Pike, Chiu-Chen Tseng, Jolanta Kupryjanczyk, Agnieszka Dansonka-Mieszkowska, Agnieszka Budzilowska, Iwona K. Rzepecka, Penelope M. Webb, on behalf of the Ovarian Cancer Association Consortium.

**Corresponding author:** Suzanne Dixon-Suen, Gynaecological Cancers Group, QIMR Berghofer Medical Research Institute, Brisbane, Australia. Email: [Suzanne.Dixon@qimrberghofer.edu.au](mailto:Suzanne.Dixon@qimrberghofer.edu.au).

**Supplementary Table 1. Studies included in the analysis.**

| **Acronym** | **Name of study/studies** | **Reference** |
| --- | --- | --- |
| AUS | Australian Ovarian Cancer Study/Australian Cancer Study (Ovarian Cancer) | 1 |
| BAV | Bavarian Ovarian Cancer Cases and Controls | 2 |
| BEL | Belgian Ovarium Cancer Study | 2 |
| DOV | Diseases of the Ovary and their Evaluation | 3 |
| GER | German Ovarian Cancer Study | 4 |
| GRR | Gilda Radner Familial Ovarian Cancer Registry | 5, 6 |
| HAW | Hawaii Ovarian Cancer Case-Control Study | 7 |
| HJO | Hannover-Jena Ovarian Cancer Study | 2 |
| HMO | Hannover-Minsk Ovarian Cancer Study | 8 |
| HOC | Helsinki Ovarian Cancer Study | 9 |
| HOP | Novel Risk Factors and Potential Early Detection Markers for Ovarian Cancer | 10 |
| HSK | Dr Horst Schmidt Kliniken | 11, 12 |
| LAX | Women's Cancer Program at the Samuel Oschin Comprehensive Cancer Institute | 13 |
| MAL | MALignant OVArian cancer | 14-16 |
| MAY | Mayo Clinic Ovarian Cancer Case-Control Study | 17, 18 |
| MCC | Melbourne Collaborative Cohort Study | 19 |
| MDA | MD Anderson Cancer Center | 13 |
| MSK | Memorial Sloan-Kettering Cancer Center | 13 |
| NCO | North Carolina Ovarian Cancer Study | 20, 21 |
| NEC | New England Case Control Study | 22, 23 |
| NJO | New Jersey Ovarian Cancer Study | 24, 25 |
| NOR | University of Bergen, Haukeland University Hospital, Norway | 26, 27 |
| NTH | Nijmegen Ovarian Cancer Study | 28, 29 |
| ORE | Oregon Ovarian Cancer Registry | 30, 31 |
| OVA | Ovarian Cancer in Alberta and British Columbia | 32 |
| POC | Polish Ovarian Cancer Study | 13 |
| POL | Polish Ovarian Cancer Case Control Study | 33 |
| PVD | Danish Pelvic Mass Study | 34, 35 |
| RMH | Royal Marsden Hospital Ovarian Cancer Study | 36 |
| SEA | Study of Epidemiology and Risk Factors in Cancer Heredity | 37 |
| SOC | Southampton Ovarian Cancer Study | 38, 39 |
| SRO | Scottish Randomised Trial in Ovarian Cancer | 40, 41 |
| STA | Family Registry for Ovarian Cancer, and Genetic Epidemiology of Ovarian Cancer | 42 |
| TOR | Familial Ovarian Tumour Study, and Health Watch | 43 |
| UCI | University California Irvine Ovarian Study | 44 |
| UKO | United Kingdom Ovarian cancer Population Study | 45 |
| UKR | UK Familial Ovarian Cancer Registry | 46 |
| USC | Los Angeles County Case-Control Studies of Ovarian Cancer | 47-49 |
| WOC | Warsaw Ovarian Cancer Study | 50 |

**Supplementary Table 2. Single nucleotide polymorphisms identified by GIANT^51^ , grouped by inclusion in our height genetic risk scores^a b^**

| **SNPs (rs numbers)** | **GRS-609** | **GRS-363** | **GRS-377** | **GRS-92** |
| --- | --- | --- | --- | --- |
| rs11799609, rs3814333, rs6694089, rs6696239, rs991967, rs3791679, rs780094, rs9309101, rs13088462, rs2581830, rs3915129, rs9841435, rs9880211, rs1996422, rs2302580, rs955748, rs26024, rs4624820, rs820848, rs314263, rs7740107, rs1055144, rs273945, rs42039, rs1036821, rs2956605, rs3763631, rs7033487, rs11049611, rs11612228, rs2306694, rs3825199, rs8756, rs11618507, rs5742915, rs975210, rs1659127, rs217181, rs8052560, rs3760318, rs3923086, rs9217, rs2074977, rs2682587, rs1326023, rs4812586, rs6061231, rs9977276 | ✓ | ✓ | ✓ | ✓ |
| rs17163588, rs3767627, rs12329133, rs7561273, rs7605699, rs992157, rs2240919, rs6762606, rs7633464, rs1812175, rs868489, rs7731703, rs9327705, rs1341278, rs1415701, rs3020418, rs3800461, rs4713902, rs12533079, rs10958476, rs17088184, rs2737220, rs4448343, rs11198820, rs11599750, rs1815314, rs17659078, rs2735469, rs2915404, rs4320932, rs4357716, rs10492364, rs1861908, rs2164968, rs12863103, rs7334755, rs129963, rs2377058, rs2070776, rs6504389, rs8067165, rs8073177, rs1535466, rs6020202 | ✓ | ✓ |  | ✓ |
| rs10779751, rs10863936, rs12120956, rs12855, rs1544196, rs212524, rs2275325, rs2811594, rs425277, rs564914, rs6600365, rs7517682, rs7544462, rs7551732, rs9428104, rs11684404, rs11687941, rs12470505, rs12474201, rs12693589, rs354196, rs4953951, rs6714546, rs6761041, rs7567851, rs7568069, rs897080, rs1546391, rs1658351, rs17806888, rs1797625, rs2633761, rs4686904, rs4974480, rs724016, rs936339, rs9835332, rs9858528, rs12513181, rs12639764, rs13113518, rs17081935, rs17556750, rs2306596, rs3958122, rs6813055, rs6838153, rs7692995, rs13177718, rs1582931, rs17410035, rs2662027, rs301901, rs32855, rs3812040, rs39623, rs422421, rs6894139, rs7701414, rs7716219, rs7733195, rs9291926, rs10948222, rs11156098, rs1155939, rs12214804, rs2145357, rs2748483, rs2763273, rs486359, rs4896582, rs6902771, rs6911389, rs6920372, rs7743622, rs9392918, rs9395264, rs12538407, rs3807931, rs552707, rs6949739, rs6952113, rs6955948, rs6974574, rs798497, rs929637, rs11783655, rs1550162, rs1599473, rs3812423, rs4733724, rs4735677, rs4875421, rs6988484, rs7007200, rs1571892, rs1576900, rs181338, rs2149163, rs7033940, rs7849585, rs7853235, rs10794175, rs10995319, rs10997979, rs12779328, rs1614303, rs1923367, rs2631676, rs291979, rs6584575, rs7069985, rs7899004, rs10767838, rs1461503, rs1945237, rs3782089, rs7112925, rs10877030, rs10880969, rs11047239, rs11616067, rs12228415, rs1420023, rs2164747, rs2856321, rs2888893, rs497273, rs7980687, rs12323101, rs12871822, rs3118905, rs3818416, rs7319045, rs7985356, rs11624136, rs1980850, rs2093210, rs7154721, rs10152739, rs16964211, rs16968242, rs316618, rs4548838, rs7162825, rs11642612, rs2326458, rs3790086, rs4785393, rs6420435, rs8058684, rs10083886, rs11867479, rs199515, rs2079795, rs2117563, rs4605213, rs8069300, rs870183, rs9766, rs11152213, rs4369779, rs11880992, rs2059877, rs4802134, rs7253628, rs7259684, rs8103992, rs1074683, rs2425163, rs2211866, rs2829941, rs2834442 | ✓ | ✓ | ✓ |  |
| rs1014987, rs12125882, rs12144094, rs1321666, rs1409156, rs17369123, rs2219320, rs4652773, rs926438, rs1367226, rs17032525, rs1864439, rs2343240, rs3791673, rs4973429, rs6751657, rs6754426, rs711245, rs994533, rs2300921, rs2596831, rs4325879, rs509035, rs7646824, rs17777628, rs6829680, rs867245, rs1004202, rs11950938, rs12153391, rs1529701, rs33852, rs4868645, rs6594336, rs6596075, rs7712162, rs12204421, rs1265097, rs17603945, rs3957165, rs648831, rs6899744, rs6919534, rs6921207, rs7774834, rs806794, rs9405356, rs2390151, rs2888877, rs7782764, rs4273857, rs6577717, rs894343, rs10119624, rs10759774, rs10962832, rs10990303, rs1742829, rs3812591, rs7870753, rs902143, rs9409082, rs11245515, rs703985, rs10766065, rs3750972, rs7126398, rs10843390, rs10859567, rs11057552, rs12820411, rs1809889, rs833706, rs1753637, rs6563199, rs1190545, rs10152591, rs10744956, rs11633371, rs2238300, rs4337252, rs7162542, rs7170986, rs731874, rs782930, rs11861084, rs12597498, rs11867943, rs1625895, rs2044124, rs2072153, rs2378870, rs8073371, rs9889755, rs10401193, rs11880124, rs17721822, rs6137287 | ✓ | ✓ |  |  |
| rs12137162, rs1325596, rs16834765, rs17113369, rs17391694, rs1935157, rs2284746, rs2298265, rs2806561, rs2815379, rs4656220, rs567401, rs6540834, rs6658763, rs6688100, rs9434723, rs11683207, rs12621643, rs12987566, rs13006748, rs13388725, rs13416119, rs2120335, rs2289195, rs2345835, rs3116168, rs4425077, rs540652, rs6435143, rs6746356, rs749234, rs7567288, rs12330322, rs2034172, rs2175513, rs2597513, rs6441170, rs6794009, rs720390, rs7652177, rs9816693, rs9825951, rs1562975, rs763318, rs7659107, rs996743, rs9993613, rs11750568, rs12186664, rs165189, rs17574650, rs2247870, rs2961830, rs2974438, rs34651, rs526896, rs7727731, rs9292468, rs12190423, rs12209223, rs1405212, rs16895130, rs17330192, rs1832871, rs310421, rs4141885, rs761391, rs932445, rs991946, rs1113765, rs17250196, rs17807185, rs2715094, rs4725061, rs6462432, rs6962887, rs6971575, rs822531, rs11779459, rs2013265, rs429433, rs568610, rs7834383, rs8180991, rs9650315, rs3132297, rs3739707, rs7027110, rs7043114, rs7466269, rs817300, rs953199, rs989393, rs10883563, rs1171615, rs4332428, rs4350272, rs915506, rs1681630, rs2237886, rs2272566, rs2510396, rs3802758, rs632124, rs6485978, rs757081, rs10748128, rs11835818, rs17122659, rs17783015, rs7971536, rs11616380, rs4883972, rs12882130, rs1950500, rs2058092, rs2781373, rs8017130, rs862034, rs11855014, rs12904334, rs17349981, rs2257011, rs2871865, rs7177711, rs7181724, rs1966913, rs2023693, rs26868, rs4843367, rs1552173, rs2072268, rs2338115, rs2854207, rs318095, rs3809790, rs4640244, rs4986172, rs584828, rs11659752, rs14062, rs692964, rs9967417, rs2123731, rs4803468, rs8102380, rs8103068, rs891088, rs17450430, rs1884897, rs2224538, rs6080830, rs7261425, rs11090631, rs7284476, rs738288 | ✓ |  | ✓ |  |
| rs10495098, rs12119525, rs12411277, rs17387330, rs2046158, rs2421992, rs4428898, rs956796, rs10048625, rs11677466, rs16859517, rs17181956, rs2278483, rs2305833, rs4344931, rs4674354, rs6544089, rs749052, rs11708412, rs13078528, rs1533269, rs16860216, rs4256170, rs11731978, rs13133465, rs16994718, rs17499117, rs4240326, rs4834927, rs6845999, rs7654571, rs961014, rs11745439, rs12055154, rs13183624, rs17075869, rs4620037, rs6887276, rs1040941, rs3828760, rs389663, rs479744, rs4895801, rs6903448, rs7745166, rs9328445, rs9443804, rs1007358, rs2188177, rs16939034, rs6999671, rs7823327, rs10820814, rs12344396, rs12347744, rs1257763, rs1329393, rs2451948, rs3927536, rs11244750, rs4746769, rs7097701, rs11236294, rs2099745, rs11107062, rs11175992, rs4326884, rs4767473, rs7299326, rs2687950, rs10140101, rs4901537, rs6571772, rs11634405, rs1348002, rs2573625, rs4246302, rs8028843, rs8042424, rs2531992, rs300039, rs3748394, rs11661645, rs12454567, rs12458127, rs2337143, rs1346490, rs6511689, rs143384, rs5757318 | ✓ |  |  |  |

GIANT, Genetic Investigation of ANthropometric Traits consortium; GRS, genetic risk score; SNP, single nucleotide polymorphism.

^a^ In our controls, minor allele frequencies for the 609 SNPs included in the GRS were consistent with those reported by GIANT.

^b^ 88 SNPs identified by GIANT had an imputation quality score (r^2^, estimated correlation between imputed and true genotype) of <0.6 in our data and were not included in any scores (rs4601530, rs209918, rs3014219, rs6691924, rs12731056, rs7534365, rs1244981, rs17038954, rs3885668, rs10460566, rs17511102, rs12615742, rs2166898, rs833152, rs6733349, rs2679184, rs13393800, rs6439168, rs11714558, rs11722554, rs6446315, rs2167645, rs11100790, rs13150868, rs3811958, rs10059761, rs12519505, rs4868126, rs1368380, rs6879260, rs163071, rs4246079, rs1047014, rs1233627, rs9404952, rs6457374, rs2857693, rs3129254, rs9456307, rs17140875, rs12669267, rs10283100, rs10972628, rs11144688, rs958225, rs10780910, rs999599, rs10817960, rs2509133, rs606452, rs10790381, rs11221442, rs10770705, rs1199734, rs6561319, rs17792664, rs12435366, rs10131337, rs8006657, rs1036477, rs17264185, rs12914466, rs16942341, rs2280470, rs3817428, rs11648796, rs2014467, rs12926008, rs1053996, rs960006, rs9929889, rs11640018, rs4243206, rs2028067, rs3169906, rs227724, rs1401795, rs1478610, rs4239020, rs888403, rs8097893, rs4072910, rs7273787, rs6085662, rs8117259, rs2057291, rs3026499, rs2413143).

**
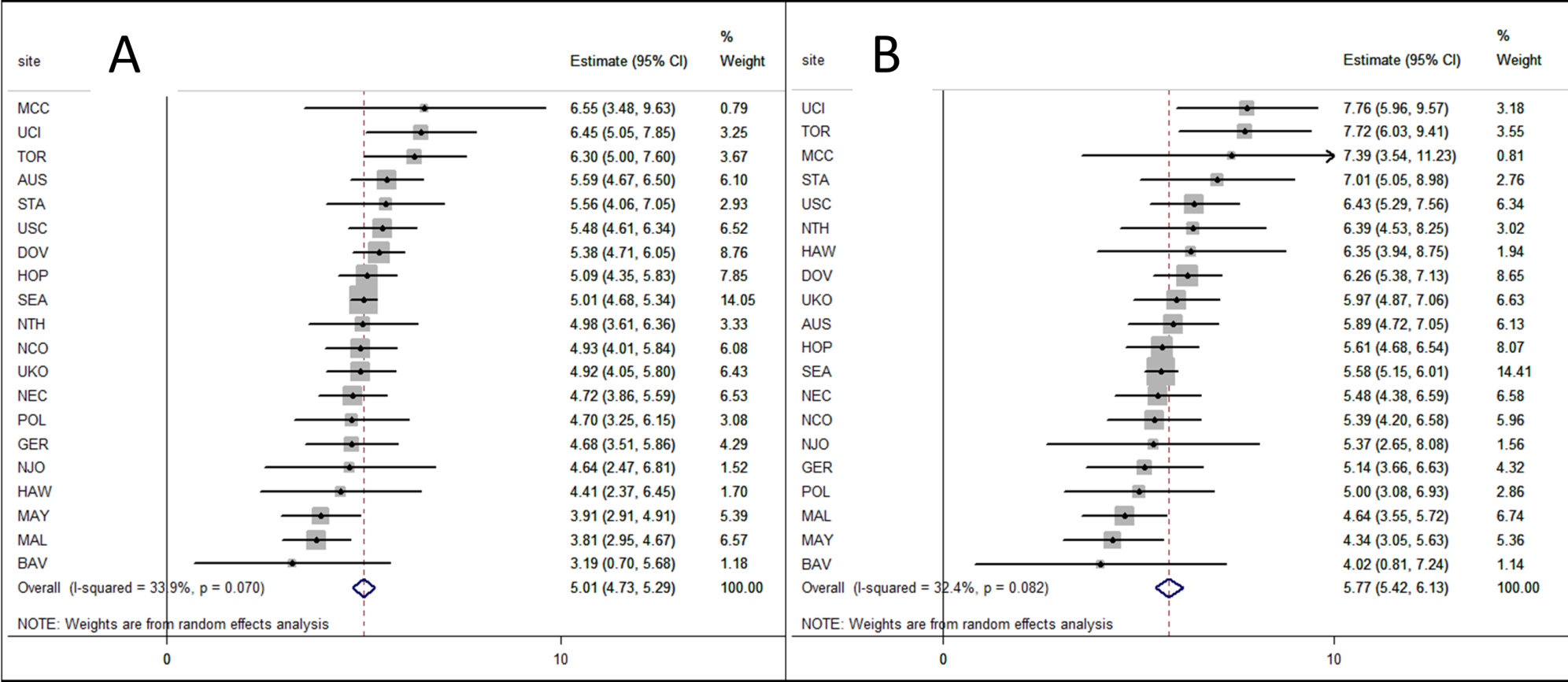

Supplementary Figure 1. Association between two genetic risk score (GRS) versions and height, by study.**

(A) GRS comprising 609 SNPs. (B) GRS comprising 363 SNPs with imputation quality scores ≥0·9.

**Supplementary Table 3. Association between potential confounders and adult height and the height genetic risk score, among controls ^a^**

| **Characteristic** | **Adult height, cm  (Mean [SD])** | ***P-*value^b^** | **GRS-609**  **Mean (SD)** | ***P-*value^b^** |
| --- | --- | --- | --- | --- |
| Age at diagnosis |  |  |  |  |
| <40 | 1.66 (0.07) |  | 17.2 (0.4) |  |
| 40-49 | 1.64 (0.07) |  | 17.2 (0.5) |  |
| 50-59 | 1.64 (0.06) |  | 17.2 (0.5) |  |
| 60-69 | 1.62 (0.06) |  | 17.2 (0.5) |  |
| ≥70 | 1.61 (0.07) | <0.0001 | 17.2 (0.5) | 0.2 |
| Number of full-term pregnancies^c^ |  |  |  |  |
| 0 | 1.64 (0.07) |  | 17.2 (0.5) |  |
| ≥1 | 1.63 (0.07) | <0.0001 | 17.2 (0.5) | 0.3 |
| Oral contraceptive use |  |  |  |  |
| Never | 1.62 (0.07) |  | 17.2 (0.5) |  |
| Ever | 1.64 (0.07) | <0.0001 | 17.2 (0.5) | 0.95 |
| *<5 years* | *1.64 (0.07)* |  | *17.2 (0.5)* |  |
| *≥ 5 years* | *1.64 (0.07)* | *<0.0001* | *17.2 (0.5)* | *0.2* |
| Attained education |  |  |  |  |
| High school or lower | 1.63 (0.07) |  | 17.3 (0.5) |  |
| Trade/college/higher education | 1.64 (0.07) | <0.0001 | 17.2 (0.5) | 0.6 |
| Age at menarche |  |  |  |  |
| <10 | 1.61 (0.07) |  | 17.2 (0.5) |  |
| 10-12 | 1.63 (0.07) |  | 17.2 (0.5) |  |
| 13-15 | 1.63 (0.07) |  | 17.2 (0.5) |  |
| ≥16 | 1.64 (0.07) | <0.0001 | 17.3 (0.5) | 0.03 |

GRS, genetic risk score; NA, not applicable; SD, standard deviation.

1. Data are summarised for case-control studies where >50% participants had data available. Height data are for a maximum of 20 studies; GRS data are for a maximum of 39 studies. We used χ^2^ statistics or analysis of variance stratified by study.
2. *P*-values are from comparisons adjusting for study. *P*-values for age (at menarche and at diagnosis) use continuous age.
3. Defined as longer than 6 months.

**Supplementary Table 4. Association between increasing height, and between increasing genetically-predicted height, per 5 cm, and risk of ovarian cancer, among women with height and confounder data.**

|  |  | **Odds Ratios (95% CI)^a^** | |
| --- | --- | --- | --- |
| **Histologic subtype^b^** | **N studies** | **from conventional analysis^c^** | **from MR analysis^d^** |
| Primary outcomes |  |  |  |
| All ovarian cancers | 16 | 1.01 (0.99-1.04) | 1.06 (1.00-1.13) |
| Invasive | 16 | 1.00 (0.98-1.03) | 1.06 (1.00-1.14) |
| Borderline | 10 | 1.08 (1.02-1.14) | 1.13 (0.99-1.29) |
| Secondary outcomes, by histologic subtype and behaviour | | | |
| Serous |  |  |  |
| High-grade | 16 | 0.98 (0.95-1.01) | 1.03 (0.95-1.11) |
| Invasive low-grade & borderline | 14 | 1.04 (0.98-1.10) | 1.17 (1.02-1.35) |
| Mucinous (invasive & borderline) | 16 | 1.08 (1.02-1.15) | 1.20 (1.04-1.39) |
| Endometrioid (invasive) | 16 | 1.02 (0.97-1.08) | 1.10 (0.96-1.25) |
| Clear cell (invasive) | 15 | 1.10 (1.02-1.19) | 1.17 (0.97-1.42) |

CI, confidence interval.

1. Odds ratios were stratified by study.
2. Includes studies with >5 cases.
3. Conventional epidemiological analysis modelled case-control status on height. Models were adjusted for parity, oral contraceptive use, education, and age at menarche, and stratified by study and 5-year age group.
4. Height predicted by a weighted 609-locus genetic risk score.

**References**

1. Merritt MA, Green AC, Nagle CM, Webb PM. Talcum powder, chronic pelvic inflammation and NSAIDs in relation to risk of epithelial ovarian cancer. Int J Cancer 2008;122:170-76.

2. Song H, Ramus SJ, Tyrer J, Bolton KL, Gentry-Maharaj A, Wozniak E, Anton-Culver H, Chang-Claude J, Cramer DW, DiCioccio R, Dork T, Goode EL, et al. A genome-wide association study identifies a new ovarian cancer susceptibility locus on 9p22.2. Nat Genet 2009;41:996-1000.

3. Rossing MA, Cushing-Haugen KL, Wicklund KG, Doherty JA, Weiss NS. Menopausal hormone therapy and risk of epithelial ovarian cancer. Cancer Epidemiol Biomarkers Prev 2007;16:2548-56.

4. Royar J, Becher H, Chang-Claude J. Low-dose oral contraceptives: protective effect on ovarian cancer risk. Int J Cancer 2001;95:370-74.

5. Jishi MF, Itnyre JH, Oakley-Girvan IA, Piver MS, Whittemore AS. Risks of cancer among members of families in the Gilda Radner Familial Ovarian Cancer Registry. Cancer 1995;76:1416-21.

6. Piver MS, Tsukada Y, Werness BA, DiCioccio RA, Whittemore AS, Ponder BA. Comparative study of ovarian cancer histopathology by registry pathologists and referral pathologists: a study by the Gilda Radner Familial Ovarian Cancer Registry. Gynecol Oncol 2000;78:166-70.

7. Goodman MT, Lurie G, Thompson PJ, McDuffie KE, Carney ME. Association of two common single-nucleotide polymorphisms in the CYP19A1 locus and ovarian cancer risk. Endocr Relat Cancer 2008;15:1055-60.

8. Bogdanova NV, Antonenkova NN, Rogov YI, Karstens JH, Hillemanns P, Dörk T. High frequency and allele-specific differences of BRCA1 founder mutations in breast cancer and ovarian cancer patients from Belarus. Clin Genet 2010;78:364-72.

9. Sarantaus L, Vahteristo P, Bloom E, Tamminen A, Unkila-Kallio L, Butzow R, Nevanlinna H. BRCA1 and BRCA2 mutations among 233 unselected Finnish ovarian carcinoma patients. Eur J Hum Genet. 2001;9:424-30.

10. Ness RB, Dodge RC, Edwards RP, Baker JA, Moysich KB. Contraception methods, beyond oral contraceptives and tubal ligation, and risk of ovarian cancer. Ann Epidemiol 2011;21:188-96.

11. du Bois A, Luck HJ, Meier W, Adams HP, Mobus V, Costa S, Bauknecht T, Richter B, Warm M, Schroder W, Olbricht S, Nitz U, et al. A randomized clinical trial of cisplatin/paclitaxel versus carboplatin/paclitaxel as first-line treatment of ovarian cancer. J Natl Cancer Inst 2003;95:1320-30.

12. Harter P, Muallem ZM, Buhrmann C, Lorenz D, Kaub C, Hils R, Kommoss S, Heitz F, Traut A, du Bois A. Impact of a structured quality management program on surgical outcome in primary advanced ovarian cancer. Gynecol Oncol 2011;121:615-19.

13. Pharoah PDP, Tsai YY, Ramus SJ, Phelan CM, Goode EL, Lawrenson K, Buckley M, Fridley BL, Tyrer JP, Shen H, Weber R, Karevan R, et al. GWAS meta-analysis and replication identifies three new susceptibility loci for ovarian cancer. Nat Genet 2013;45:362-70.

14. Huusom LD, Frederiksen K, Hogdall EV, Glud E, Christensen L, Hogdall CK, Blaakaer J, Kjaer SK. Association of Reproductive Factors, Oral Contraceptive Use and Selected Lifestyle Factors with the Risk of Ovarian Borderline Tumors: A Danish Case-control Study. Cancer Causes Control 2006;17:821-29.

15. Glud E, Kjaer SK, Thomsen BL, Hogdall C, Christensen L, Hogdall E, Bock JE, Blaakaer J. Hormone therapy and the impact of estrogen intake on the risk of ovarian cancer. Arch Intern Med 2004;164:2253-59.

16. Soegaard M, Jensen A, Hogdall E, Christensen L, Hogdall C, Blaakaer J, Kjaer SK. Different risk factor profiles for mucinous and nonmucinous ovarian cancer: results from the Danish MALOVA study. Cancer Epidemiol Biomarkers Prev 2007;16:1160-66.

17. Goode EL, Maurer MJ, Sellers TA, Phelan CM, Kalli KR, Fridley BL, Vierkant RA, Armasu SM, White KL, Keeney GL, Cliby WA, Rider DN, et al. Inherited Determinants of Ovarian Cancer Survival. Clin Cancer Res 2010;16:995-1007.

18. Kelemen LE, Sellers TA, Schildkraut JM, Cunningham JM, Vierkant RA, Pankratz VS, Fredericksen ZS, Gadre MK, Rider DN, Liebow M, Goode EL. Genetic variation in the one-carbon transfer pathway and ovarian cancer risk. Cancer Res 2008;68:2498-506.

19. Giles G, English D. The Melbourne Collaborative Cohort Study. IARC Sci Publ 2002;156:69-70.

20. Schildkraut JM, Iversen ES, Wilson MA, Clyde MA, Moorman PG, Palmieri RT, Whitaker R, Bentley RC, Marks JR, Berchuck A. Association between DNA damage response and repair genes and risk of invasive serous ovarian cancer. PloS One. 2010;5:e10061.

21. Schildkraut JM, Moorman PG, Bland AE, Halabi S, Calingaert B, Whitaker R, Lee PS, Elkins-Williams T, Bentley RC, Marks JR, Berchuck A. Cyclin E overexpression in epithelial ovarian cancer characterizes an etiologic subgroup. Cancer Epidemiol Biomarkers Prev 2008;17:585-93.

22. Terry KL, De Vivo I, Titus-Ernstoff L, Shih M-C, Cramer DW. Androgen receptor cytosine, adenine, guanine repeats, and haplotypes in relation to ovarian cancer risk. Cancer Res 2005;65:5974-81.

23. Terry KL, Tworoger SS, Goode EL, Gates MA, Titus-Ernstoff L, Kelemen LE, Sellers TA, Hankinson SE, Cramer DW. MTHFR polymorphisms in relation to ovarian cancer risk. Gynecol Oncol 2010;119:319-24.

24. Bandera EV, King M, Chandran U, Paddock LE, Rodriguez-Rodriguez L, Olson SH. Phytoestrogen consumption from foods and supplements and epithelial ovarian cancer risk: a population-based case control study. BMC Womens Health 2011;11:40-48.

25. Chandran U, Bandera EV, Williams-King MG, Paddock LE, Rodriguez-Rodriguez L, Lu SE, Faulkner S, Pulick K, Olson SH. Healthy eating index and ovarian cancer risk. Cancer Causes Control 2011;22:563-71.

26. Salvesen HB, Carter SL, Mannelqvist M, Dutt A, Getz G, Stefansson IM, Raeder MB, Sos ML, Engelsen IB, Trovik J, Wik E, Greulich H, et al. Integrated Genomic Profiling of Endometrial Carcinoma Associates Aggressive Tumors with Indicators of PI3 Kinase Activation. Proc Natl Acad Sci U S A 2009;106:4834-39.

27. Etemadmoghadam D, deFazio A, Beroukhim R, Mermel C, George J, Getz G, Tothill R, Okamoto A, Raeder MB, Group AS, Harnett P, Lade S, et al. Integrated Genome-Wide DNA Copy Number and Expression Analysis Identifies Distinct Mechanisms of Primary Chemoresistance in Ovarian Carcinomas. Clin Cancer Res 2009;15:1417-27.

28. Bolton KL, Tyrer J, Song H, Ramus SJ, Notaridou M, Jones C, Sher T, Gentry-Maharaj A, Wozniak E, Tsai YY, Weidhaas J, Paik D, et al. Common variants at 19p13 are associated with susceptibility to ovarian cancer. Nat Genet 2010;42:880-84.

29. Goode EL, Chenevix-Trench G, Song H, Ramus SJ, Notaridou M, Lawrenson K, Widschwendter M, Vierkant RA, Larson MC, Kjaer SK, Birrer MJ, Berchuck A, et al. A genome-wide association study identifies susceptibility loci for ovarian cancer at 2q31 and 8q24. Nat Genet 2010;42:874-79.

30. Pejovic T, Pande NT, Mori M, Mhawech-Fauceglia P, Harrington C, Mongoue-Tchokote S, Dim D, Andrews C, Beck A, Tarumi Y, Djilas J, Cappuccini F, et al. Expression profiling of the ovarian surface kinome reveals candidate genes for early neoplastic changes. Translational oncology 2009;2:341-49.

31. Pejovic T, Yates JE, Liu HY, Hays LE, Akkari Y, Torimaru Y, Keeble W, Rathbun RK, Rodgers WH, Bale AE, Ameziane N, Zwaan CM, et al. Cytogenetic instability in ovarian epithelial cells from women at risk of ovarian cancer. Cancer Res 2006;66:9017-25.

32. Earp MA, Kelemen LE, Magliocco AM, Swenerton KD, Chenevix-Trench G, Lu Y, Hein A, Ekici AB, Beckmann MW, Fasching PA, Lambrechts D, Despierre E, et al. Genome-wide association study of subtype-specific epithelial ovarian cancer risk alleles using pooled DNA. Hum Genet 2014;133:481-97.

33. Garcia-Closas M, Brinton LA, Lissowska J, Richesson D, Sherman ME, Szeszenia-Dabrowska N, Peplonska B, Welch R, Yeager M, Zatonski W, Chanock SJ. Ovarian cancer risk and common variation in the sex hormone-binding globulin gene: a population-based case-control study. BMC cancer. 2007;7:60-66.

34. Høgdall E, Fung ET, Christensen IJ, Yip C, Nedergaard L, Engelholm SA, Risum S, Petri AL, Lundvall L, Lomas L, Høgdall C. Proteomic biomarkers for overall and progression-free survival in ovarian cancer patients. Proteomics Clin Appl 2010;4:940-52.

35. Risum S. Standardized FDG uptake as a prognostic variable and as a predictor of incomplete cytoreduction in primary advanced ovarian cancer. Acta Oncol 2011;50:415-19.

36. Stratton JF, Gayther SA, Russell P, Dearden J, Gore M, Blake P, Easton D, Ponder BAJ. Contribution of BRCA1 Mutations to Ovarian Cancer. N Engl J Med 1997;336:1125-30.

37. Song H, Ramus SJ, Quaye L, DiCioccio RA, Tyrer J, Lomas E, Shadforth D, Hogdall E, Hogdall C, McGuire V, Whittemore AS, Easton DF, et al. Common variants in mismatch repair genes and risk of invasive ovarian cancer. Carcinogenesis 2006;27:2235-42.

38. Baxter SW, Choong DYH, Eccles DM, Campbell IG. Transforming growth factor beta receptor 1 polyalanine polymorphism and exon 5 mutation analysis in breast and ovarian cancer. Cancer Epidemiol Biomarkers Prev 2002;11:211-14.

39. Morland SJ, Jiang X, Hitchcock A, Thomas EJ, Campbell IG. Mutation of galactose-1-phosphate uridyl transferase and its association with ovarian cancer and endometriosis. Int J Cancer 1998;77:825-27.

40. Marsh S, Paul J, King CR, Gifford G, McLeod HL, Brown R. Pharmacogenetic Assessment of Toxicity and Outcome After Platinum Plus Taxane Chemotherapy in Ovarian Cancer: The Scottish Randomised Trial in Ovarian Cancer. J Clin Oncol 2007;25:4528-35.

41. Vasey PA, Jayson GC, Gordon A, Gabra H, Coleman R, Atkinson R, Parkin D, Paul J, Hay A, Kaye SB, Scottish Gynaecological Cancer Trials G, On behalf of the Scottish Gynaecological Cancer Trials G. Phase III randomized trial of docetaxel-carboplatin versus paclitaxel-carboplatin as first-line chemotherapy for ovarian carcinoma. J Natl Cancer Inst 2004;96:1682-91.

42. McGuire V, Felberg A, Mills M, Ostrow KL, DiCioccio R, John EM, West DW, Whittemore AS. Relation of contraceptive and reproductive history to ovarian cancer risk in carriers and noncarriers of BRCA1 gene mutations. Am J Epidemiol 2004;160:613-18.

43. Zhang S, Royer R, Li S, McLaughlin JR, Rosen B, Risch HA, Fan I, Bradley L, Shaw PA, Narod SA. Frequencies of BRCA1 and BRCA2 mutations among 1,342 unselected patients with invasive ovarian cancer. Gynecol Oncol 2011;121:353-57.

44. Ziogas A, Gildea M, Cohen P, Bringman D, Taylor TH, Seminara D, Barker D, Casey G, Haile R, Liao S-Y, Thomas D, Noble B, et al. Cancer Risk Estimates for Family Members of a Population-based Family Registry for Breast and Ovarian Cancer. Cancer Epidemiol Biomarkers Prev 2000;9:103-11.

45. Balogun N, Gentry-Maharaj A, Wozniak EL, Lim A, Ryan A, Ramus SJ, Ford J, Burnell M, Widschwendter M, Gessler SF, Gayther SA, Jacobs IJ, et al. Recruitment of newly diagnosed ovarian cancer patients proved challenging in a multicentre biobanking study. J Clin Epidemiol 2011;64:525-30.

46. Ramus SJ, Harrington PA, Pye C, DiCioccio RA, Cox MJ, Garlinghouse-Jones K, Oakley-Girvan I, Jacobs IJ, Hardy RM, Whittemore AS, Ponder BAJ, Piver MS, et al. Contribution of BRCA1 and BRCA2 mutations to inherited ovarian cancer. Hum Mutat 2007;28:1207-15.

47. Pike MC, Pearce CL, Peters R, Cozen W, Wan P, Wu AH. Hormonal factors and the risk of invasive ovarian cancer: a population-based case-control study. Fertil Steril 2004;82:186-95.

48. Ness RB, DW C, Goodman MT, Kruger Kjaer S, Mallin K, Mosgaard BJ, Purdie D, Risch HA, Vergona R, Wu AH. Infertility, fertility drugs and ovarian cancer: A pooled analysis of case-control studies. Am J Epidemiol 2002;155:217-24.

49. Wu AH, Pearce CL, Tseng CC, Templeman C, Pike MC. Markers of inflammation and risk of ovarian cancer in Los Angeles County. Int J Cancer 2009;124:1409-15.

50. Dansonka-Mieszkowska A, Kluska A, Moes J, Dabrowska M, Nowakowska D, Niwinska A, Derlatka P, Cendrowski K, Kupryjanczyk J. A novel germline PALB2 deletion in Polish breast and ovarian cancer patients. BMC Med Genet 2010;11:20-28.

51. Wood AR, Esko T, Yang J, Vedantam S, Pers TH, Gustafsson S, Chu AY, Estrada K, Luan Ja, Kutalik Z, Amin N, Buchkovich ML, et al. Defining the role of common variation in the genomic and biological architecture of adult human height. Nat Genet 2014;46:1173-86.
